# Diameter-driven crossover in resistive behaviour of heavily doped self-seeded germanium nanowires

**DOI:** 10.3762/bjnano.7.119

**Published:** 2016-09-13

**Authors:** Stephen Connaughton, Maria Koleśnik-Gray, Richard Hobbs, Olan Lotty, Justin D Holmes, Vojislav Krstić

**Affiliations:** 1 School of Physics, AMBER@CRANN, Trinity College Dublin, College Green, Dublin 2, Republic of Ireland; 2 Chair for Applied Physics, Department of Physics, Friedrich-Alexander-University Erlangen-Nürnberg (FAU), Staudtstraße 7, 91058 Erlangen, Germany; 3 Materials Chemistry & Analysis Group, Department of Chemistry, University College Cork, Cork, Republic of Ireland

**Keywords:** diameter-dependence, germanium, nanowire, resistivity, self-seeded

## Abstract

The dependence of the resistivity with changing diameter of heavily-doped self-seeded germanium nanowires was studied for the diameter range 40 to 11 nm. The experimental data reveal an initial strong reduction of the resistivity with diameter decrease. At about 20 nm a region of slowly varying resistivity emerges with a peak feature around 14 nm. For diameters above 20 nm, nanowires were found to be describable by classical means. For smaller diameters a quantum-based approach was required where we employed the 1D Kubo–Greenwood framework and also revealed the dominant charge carriers to be heavy holes. For both regimes the theoretical results and experimental data agree qualitatively well assuming a spatial spreading of the free holes towards the nanowire centre upon diameter reduction.

## Findings

Semiconducting nanowires are in the focus of research due to their potential applications in electronics and optics [1–9]. Germanium nanowires (Ge NWs) are of particular interest as they provide the prospect for quantum-related phenomena associated with one-dimensional (1D) confinement already at diameters of tens of nm [10], or determining their electronic properties by surface doping [11].

Among different synthetic routes for obtaining Ge NWs [12–13], the novel self-seeding mechanism is of special interest [14–15]. The main advantage of this method is the elimination of dopant incorporation from the metal nanoparticle catalysts [11–13]. It was demonstrated that by selecting the synthesis conditions, the degree of surface-doping in the NW can be controlled [11]. Even heavy doping close to or at the degeneracy level can be achieved, rendering the semiconducting NW quasi-metallic [11]. Approaching degenerate doping, therefore, provides opportunity to produce NWs adequate for both nanoscaled semiconductor conduction-channel and source- and drain-components. Ultimately, these two doping states could be realised within the same NW in different sections. In perspective of the continued successful miniaturisation of electronic devices it is therefore essential to elucidate the diameter dependence of the resistivity of heavily/degenerately doped self-seeded Ge NWs.

**Figure 1:**
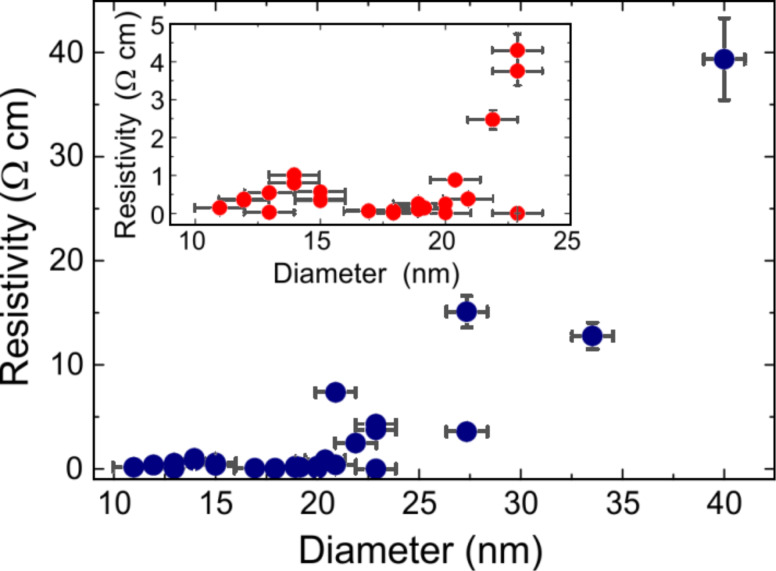
Resistivity of nanowires with 40 to 11 nm diameter. The resistivity decreases by two orders of magnitude with reducing diameter. In the range of 27 to 20 nm an increased scattering of the resistivity values is observed. Below 20 nm the resistivity variation is significantly smaller. Inset: Data for NWs with diameter below 25 nm. At around 14 nm a peak-like feature is found.

For our study we chose heavily doped self-seeded Ge NWs predominantly having the same crystallographic direction [11,14–15]. Individual NWs were transferred onto 300 nm thermally grown SiO_2_ substrates and contacted lithographically in four-point probe configuration [11]. Electrical characterization was carried out at ambient conditions. The geometry of each NW device (diameter size and channel length) was determined by electron microscopy [11,16]. From this the NW resistivity was extracted as function of diameter (Figure 1).

Upon NW diameter reduction, first the resistivity revealed a rapid drop by two orders of magnitude, followed by a region of weakly varying resistivity below 20 nm diameter. Prior to this region, an increased scattering of the resistivity values was observed coinciding with the NW diameter reaching the excitonic Bohr radius (≈24 nm [10]), indicating the approach to the quantum regime. Furthermore, a peak-like feature was found at around 14 nm diameter (inset Figure 1). In comparison, lightly doped semiconducting NWs were reported to exhibit a strong increase of resistivity with decreasing diameter [17].

**Figure 2:**
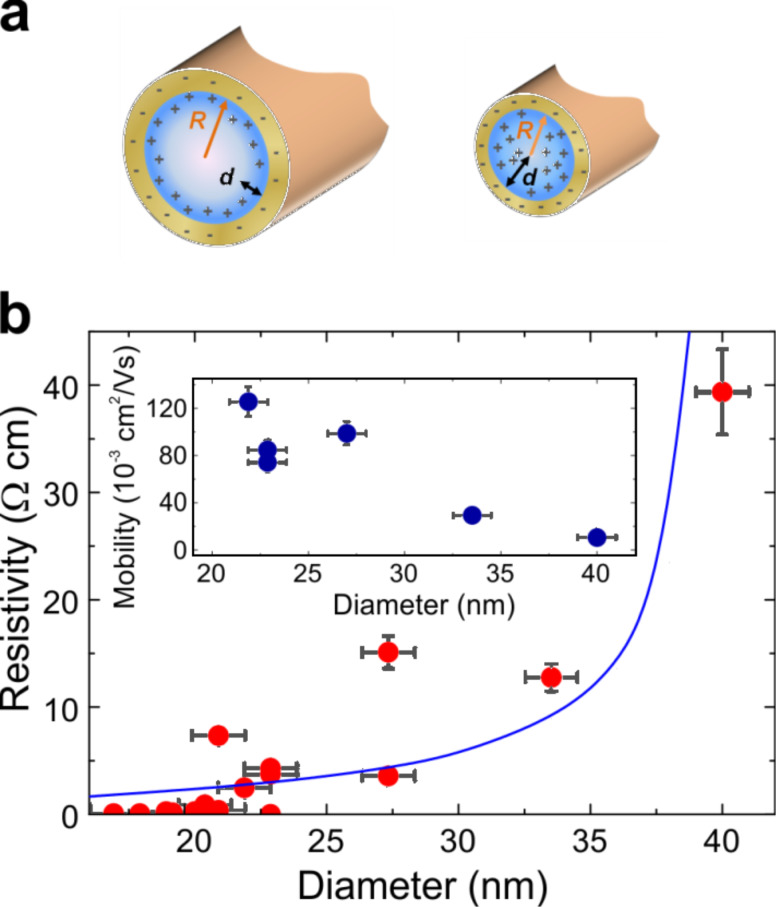
(a) Schematic diagram of the volume in which holes are predominantly located for (left) a wire with a diameter above ≈20 nm and (right) for smaller diameters. The yellow region at the outside represents the nanowire shell. Dark-blue area: region of free holes near to the core/shell interface. Light-coloured area: region devoid of free holes. Trapped electrons and free holes are schematically depicted by (−) and (+), respectively. (b) Comparison between theoretical model and experimental resistivity values for diameters larger than 22 nm. The model (blue line) follows qualitatively the observed experimental data. For smaller diameters a systematic deviation of the data is found indicating the limit of the validity of the model for these diameters. Inset: Calculated mobility values after Equation 2.

To describe our findings, we first recall that in self-seeded Ge NWs the majority charge carriers are free holes whose concentration depends on the number of charge traps at the NW core/shell interface [11,18]. In particular, for larger diameter NWs those free holes will be predominantly located in a space-charge region of width *d* near the interface [19–21] which extends towards the NW centre (schematic in Figure 2a). That is, there will be a central region along the NW axis devoid of free charge carriers. The width *d* can be calculated by solving the Poisson equation in cylindrical coordinates [20–21] and for simplicity assuming a constant free-hole concentration *n*_h_. One finds the expression [22]

[1]
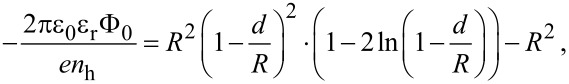


where Φ_0_ is the electrostatic potential at the core/shell interface, ε_0_ is the vacuum permittivity, ε_r_ the dielectric constant of germanium, and *e* the elementary charge.

The confinement of free holes into the space-charge region close to the NW surface only, however, cannot remain for all diameters. The available volume for the free holes and the number of charge-traps at the core/shell interface scale with NW diameter. Therefore, with decreasing diameter the holes will extend further towards the NW axis and eventually *d* will become comparable to the NW radius *R* which we find in our calculation to occur for a diameter of about 18 nm (Supporting Information File 1).

We first address the data in the region with the largest resistivity variation (diameters ≥ 20 nm). Here we presume *d* << *R* and a classical description to be sufficient. Assuming that the number of holes is the same as the number of charge traps, we get for the free-hole concentration *n*_h_ = 2η_ct_*R*·(2*Rd* – *d*^2^)^−1^ with η_ct_ being the charge trap density per cm^2^. The NW resistivity can be therefore written as (Supporting Information File 1)

[2]
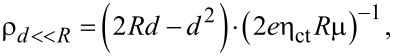


where µ is the free hole mobility. Taking Φ_0_ = 0.3 eV [22], ε_r_ = 16 [23] and η_ct_ = 10^13^ cm^−2^ [11] we can calculate from Equation 2 the corresponding mobility values (inset Figure 2b) which range from 10 to 120 × 10^−3^ cm^2^ V^−1^ s^−1^. Interestingly, the mobility increases with reducing diameter, in contrast to lightly doped semiconductor NWs [17].

Fitting the mobility data by a linear function and inserting it into Equation 2 we find resistivity values which are in good agreement with our data for NWs down to 22 nm diameter (Figure 2b) (showing consistency with our assumption *d* << *R* for nanowires ≥ 18 nm diameter; Supporting Information File 1). Below 22 nm, however, the resistivity values systematically stay well beneath our theoretical curve indicating that a classical description is insufficient. This is further corroborated by the observation of an increased scattering of the resistivity values around 22 nm (cf. Figure 1). Both findings point towards a crossover from a classical to a quantum-determined behaviour of the charge-carriers in this diameter range. Therefore, for NW diameters below 20 nm we now employ a quantum formalism taking into account that the free holes are distributed across the entire NW diameter (*d* = *R*). We consider the simple model of a NW being represented by a cylindrical well with infinite potential walls. It is shown below that despite this simplification we find qualitative correspondence with our experimental findings.

Within this approach using the parameters of bulk germanium we determined the positions of the heavy and light hole sub-band minima along with the corresponding density of states (Supporting Information File 1). The density of states for heavy holes was found to dominate and therefore these can be considered as the main carrier type. The average sub-band bottom spacing compares to the thermal energy at room temperature (Supporting Information File 1) suggesting that a description within the 1D Kubo–Greenwood framework is valid [24]. These findings also demonstrate that indeed at diameters ≤ 20 nm the confinement is not negligible and therefore the quantum regime is entered.

As dominating resistivity contributions, we considered phonon and (remote) Coulomb scattering from the charge traps at the core/shell interface (Supporting Information File 1). Surface roughness scattering was neglected within our diameter range, as previous reports on Si NWs suggest that only at diameters below 10 nm a sizable contribution is expected [24–25]. We further assumed the density of surface states to be constant.

**Figure 3:**
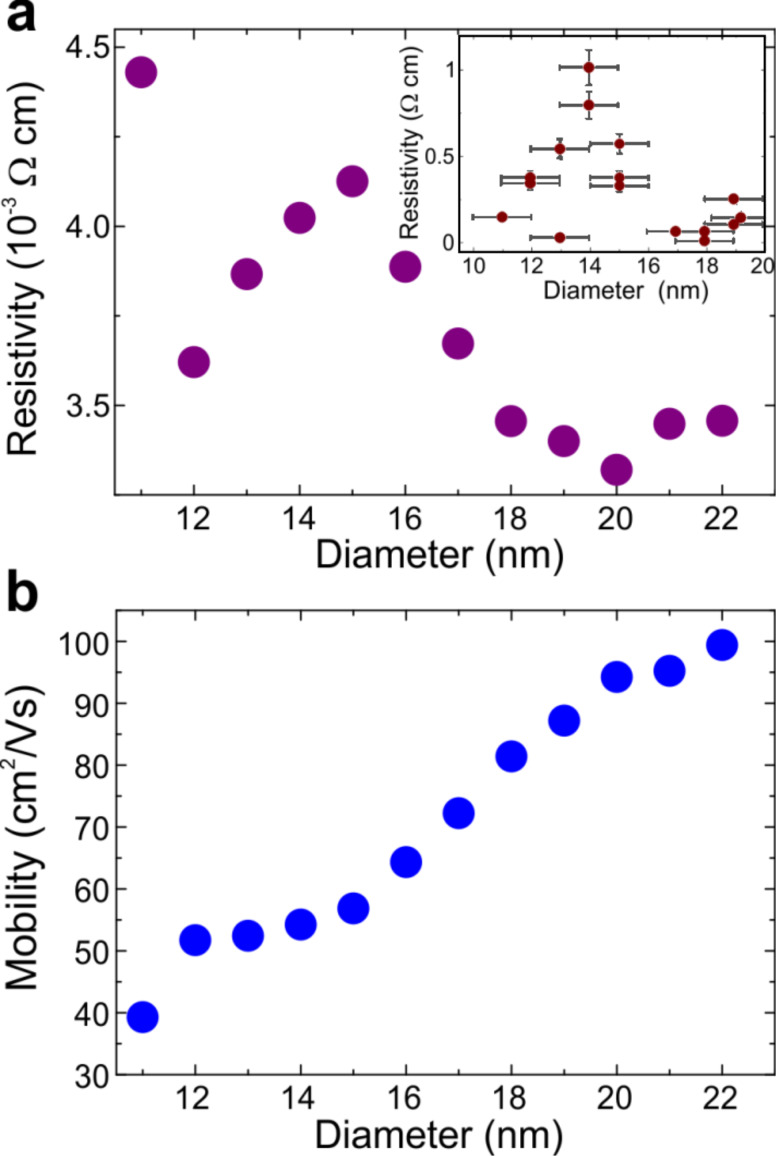
(a) Calculated nanowire resistivity from 22 to 11 nm diameter. Around 14 nm a peak-feature is found in agreement with the experimental data. At 11 nm diameter, the sharp increase of the resistivity is due to the shift of the Fermi-energy below the third heavy-hole sub-band (Supporting Information File 1). (b) Nanowire mobility calculated for the same diameter range as in (a). After an initial decrease, the mobility reveals a plateau-like region from about 12 to 15 nm.

For diameters between 11 and 22 nm, the NW resistivity and mobility values were calculated (Figure 3). A peak feature in resistivity at around 14 nm was found, in similarity to the experimental data (inset Figure 3a). The calculated mobility values decrease monotonically, developing a plateau between 12 and 15 nm diameter. This can be related to the fact that the Fermi level rises rapidly with these diameters as it is pinned by the large density of states at the bottom of sub-bands involved (Supporting Information File 1) and thus is responsible for the peak feature: The carrier concentration increases with diameter reduction (acting to lower the resistivity), in opposition to the decreasing mobility. That is, when the mobility is constant, the concentration increase dominates and the resistivity reduces as the diameter is lowered. For other diameters the mobility reduction counterbalances the concentration increase leading to a resistivity augmentation with decreasing diameter. Therefore the change from mobility- to carrier-concentration-dominated resistivity leads to the peak-feature observed. Also, the appearance of such a peak is only expected in thin nanowires (sufficiently confined electronic system) with significant interface/surface state doping.

Although the position of the peak-like feature at 14 nm is well qualitatively reproduced, there is less quantitative agreement: For the lowest value of 9 × 10^−3^ Ω cm for a 19 nm diameter NW, the estimated 3.4 × 10^−3^ Ω cm differs only by a factor of three. However, for the top of the peak feature, the calculated 4.2 × 10^−3^ Ω cm is underestimated by more than two orders of magnitude. We attribute this discrepancy to the simplifications used. The infinite potential well approximation (rather than a self-consistent solution to the Schrödinger and Poisson equations) requires the carrier wavefunctions to be strictly zero at the cylinder surface which in turn results in an underestimate of the charge carrier density within the NW surface region. This influences the actual Coulomb-scattering contribution to the resistivity as the Coulomb-scattering amplitude between any two sub-bands is strongly dependent on the NW diameter (Supporting Information File 1). While for all diameters the scattering amplitude principally increases when approaching the core/shell interface, within larger diameter NWs the difference for carriers in the centre and close to the surface augments significantly. More specifically, this increase is predominantly due to the rapid change of the scattering amplitude in the NW centre, while close to the surface the amplitude varies much less for different diameters. For example, the scattering amplitude within the centre region of a 12 nm diameter nanowire is more than an order of magnitude higher compared to a NW of 17 nm diameter. In contrast, the amplitudes close to the NW surfaces differ only by a few ten % (Supporting Information File 1).

## Supporting Information

File 1 Details on theoretical calculations.
