# Supplementary material for: Diameter-driven crossover in resistive behaviour of heavily doped self-seeded germanium nanowires
Source: Beilstein J Nanotechnol. 2016 Sep 13;7:1284–8. doi: 10.3762/bjnano.7.119 (PMC5082314; doi:10.3762/bjnano.7.119)
Supplement: File 1 — Details on theoretical calculations. [file Beilstein_J_Nanotechnol-07-1284-s001.pdf]

Supporting Information
for
Diameter-driven crossover in resistive behaviour of heavily doped self-seeded germanium nanowires

Stephen Connaughton[1], Maria Koleśnik-Gray[2], Richard Hobbs[3], Olan Lotty[3], Justin D. Holmes[3], and Vojislav Krstić[*1,2]

Address: [1]School of Physics, AMBER@CRANN, Trinity College Dublin, College Green, Dublin 2, Republic of Ireland, [2]Chair for Applied Physics, Department of Physics, Friedrich-Alexander-University Erlangen-Nürnberg (FAU), Staudtstraße 7, 91058 Erlangen, Germany and [3]Materials Chemistry & Analysis Group, Department of Chemistry, University College Cork, Cork, Republic of Ireland

Email: Vojislav Krstić - vojislav.krstic@fau.de

*Corresponding author

Details on theoretical calculations

<u>Number of free holes in nanowire</u>

The number of free holes in the core of the nanowire will depend on the number of charge traps at the core/shell interface. We assume for simplicity that the total number of carriers from the surface states, N_{ss}, is equal to the total number of occupied surface states, given by:

$$N_{ss} = 2\pi l R n_{ss}, \qquad \text{(S1)}$$

where R is the radius of the nanowire, n_{ss} is the density of occupied surface states and l is the length of the wire. Equation S1 shows that the number of carriers is linearly proportional to the radius if the density of surface states is constant. We

assume that the surface state density is constant for all wires grown with the same growth conditions. The resistivity depends not on the carrier number, as given by Equation S1, but on the carrier concentration. To find the carrier concentration we shall need to calculate the volume of the region to which the carriers are confined.

Regime 1: $R \gg d$

The free holes in the germanium core are attracted to the electrons trapped in the shell and consequently for large diameter wires exist predominantly in a region near the interface called the *space-charge region* [1, 2]. The depth d to which the space-charge region extends into the sample is found by solving Poisson's equation [2]. Assuming that the space-charge region has a constant charge density n_{sc}, it can be shown that for a cylindrical geometry [3]:

$$-\frac{2\pi\epsilon_r\epsilon_0\phi_0}{en_{sc}} = R^2\left(1-\frac{d}{R}\right)^2 \times \left(1-2\ln\left(1-\frac{d}{R}\right)\right) - R^2, \tag{S2}$$

where ϕ_0 is the electrostatic potential at the surface, ϵ_0 is vacuum permittivity, ϵ_r is the dielectric constant of germanium, and e is the charge of the electron. The carrier concentration will fall off gradually away from the surface, and there will not be a sharp distinction between the occupied space charge region and the empty centre of the nanowire. For this reason Equation S2 is only valid when $R \gg d$.

The volume of the space-charge region is the volume of the whole nanowire minus the inner cylindrical region with no charge:

$$V_{sc} = \pi R^2 l - \pi(R-d)^2 l \tag{S3}$$

and thus we have for the carrier concentration in the space charge region:

$$n_{sc} = \frac{N_{SS}}{V_{SC}} = \frac{2n_{SS}R}{2Rd-d^2}, \tag{S4}$$

where we have again assumed that there is a constant carrier concentration over some distance d. Finally, by multiplying by the charge of the electron and the mobility, μ, we can find the dependence of the resistivity, ρ, on the diameter:

$$\rho = \frac{1}{n_{sc}e\mu} = \frac{2Rd-d^2}{2n_{SS}Re\mu}. \tag{S5}$$

Regime 2: $\boldsymbol{R} \approx \boldsymbol{d}$

Wavefuction of the Charge Carriers

To calculate the mobility with the Kubo–Greenwood formula we first need to calculate the momentum relaxation times for different scattering processes. For this we require the wavefunction of the carriers in the wire. We model the nanowire as an infinite cylindrical well, assuming that the mobile holes are present throughout the entire wire volume. The advantage of this approach is that it allows us to solve for the wavefunction analytically. In cylindrical coordinates ($r = (r, \theta, z)$) the wavefuction, $\Psi(\boldsymbol{r})$ can be decomposed into its radial, $R(r)$, angular, $\Theta(\theta)$, and longitudinal, $Z(z)$, parts [8]:

$$\Psi(r) = R(r)\Theta(\theta)Z(z) \tag{S6}$$

For our calculations we only require the radial part due to cylindrical symmetry of the system. The radial part of the wavefunction is given by [8]:

$$R_{mn}(r) = R_i(r) = \frac{\sqrt{2}}{R J_{m+1}(j_{mn})} J_m\left(\frac{j_{mn}}{R} r\right), \tag{S7}$$

where $m = 0, \pm 1, \pm 2 \ldots$, $n = 1,2,3 \ldots$, $J_m$is the *m*-th Bessel function of the first kind and j_{mn} is the *n*-th positive zero of the *m*-th Bessel function. Each possible pair of values for m and n corresponds to a different subband. We will label each subband with a single index, i, instead of with m and n for clarity in later equations. We note that the assumption of an infinite potential well leads to parabolic bands. However, in low dimensional structures the bands are no longer well described as parabolic which can be accounted for by a nonparabolic correction term, α. We took the value of α to be 0.7 eV^{-1} for heavy holes and 0.2 eV^{-1} for light holes, as has previously been calculated for germanium nanowires [10].

The number of charge carriers in a nanowire can be calculated by integrating the density of states, $g(E)$, over energy up to the Fermi level, E_f:

$$n = \int_{-\infty}^{E_f} g(E) dE. \tag{S8}$$

This expression is only strictly valid at 0 K, but can be used if the Fermi level does not change by a significant amount as the sample is heated to room temperature, which should be the case for our quasi metallic nanowires [11]. Inserting Equation S4 for n and $g(E)$ (see below) into Equation S8 allows us to find the Fermi level as a function of diameter and the density of surface states.

<u>Density of States and Expectation Value of Potential Energy</u>

We calculate the density of states in one dimension starting from the non-parabolic bands. In this the following expressions hold[1]

$$k(E) = \hbar^{-1}\sqrt{2m^*[E - E_i^{(Min,P)} + \alpha(E - U_i)^2}$$

$$v_i(E) = \frac{1}{\hbar}\frac{dE}{dk} = \frac{[1+2\alpha(E-U_i)]^{-1}\hbar k(E)}{m^*} \tag{S9}$$

$$g_m^{(NP)} = \frac{\Theta\left(E - E_i^{(Min,NP)}\right)2g}{\pi\hbar v_i(E)},$$

where $v_i(E)$ is the group velocity of the carrier in subband i at energy E and g is the valley degeneracy. It should be noted that these expressions are valid for either the conduction band or the valence band. In the case of the valence band there are no valleys and so g=1. Furthermore, the effective mass will no longer be constant as the bands are no longer parabolic. The effective mass that appears in the expressions above is the effective mass for the parabolic band.

In these equations also the term U_i appears which is the expectation value of the potential energy for a given subband [4, 9], $U_i = \int \Psi_i^*(r)U\Psi_i(r)$. Due to the fact that we are setting the potential inside the wire to be 0 at all points the term U_i vanishes everywhere, simplifying the calculation.

<u>Hole-Phonon Scattering</u>

Charge carriers can be scattered by either acoustic or optical phonons. Acoustic phonons have little or no momentum at the Brillouin zone centre, in contrast to optical phonons [11]. The momentum relaxation time for a hole in the subband i due to scattering from an acoustic phonon is given by [5]:

$$\frac{1}{\tau_i^{(AC)}} = \frac{\pi E_1^2 k_B T}{\hbar \rho_{Ge} u^2}\sum_{i'} g_{i'}(E)F_{ii'}, \tag{S10}$$

where E_1 is the average acoustic deformation potential which, ρ_{Ge} is the density of germanium, u is the speed of sound in germanium, $g_{i'}(E)$ is the density of states in

[1] *cf.* S. Jin, M.V. Fischetti, T. Tang, Modeling of electron mobility in gated silicon nanowires at room temperature: Surface roughness scattering, dielectric screening, and band nonparabolicity, Journal of Applied Physics, 102 (2007) 083715-083714; A. Godoy, Z. Yang, U. Ravaioli, F. Gámiz, Effects of nonparabolic bands in quantum wires, Journal of Applied Physics, 98 (2005) 013702-013705.

the sub-band i', and $F_{ii'}$ is the so called *form factor* for a one dimensional wire, given by [5]:

$$F_{ii'} = \frac{1}{2\pi}\int_0^\infty drrR_i^2(r)R_{i'}^2(r). \tag{S11}$$

The momentum relaxation time associated with scattering from optical phonons is given by [5]:

$$\frac{1}{\tau_i^{(op)}(E)} = \frac{\pi(DK)^2}{2\rho_{Ge}\omega_{op}}\sum_{i'} g_{i'}\left(E \pm \hbar\omega_{op}\right) \times \frac{F_{ii'}\left(1-f\left(E\pm\hbar\omega_{op}\right)\right)}{1-f(E)}\left(N_{op} + \frac{1}{2} \mp \frac{1}{2}\right), \tag{S12}$$

where DK is the average optical deformation potential, $\hbar\omega_{op}$ is the energy of an optical phonon, $f(E)$ is the Fermi–Dirac distribution, N_{op} is the phonon number, determined by $\left(exp\left(\frac{k_BT}{\hbar\omega_{op}}\right) - 1\right)^{-1}$ [12], and the $\pm$ signs account for forward and backward scattering, both of which are included in the calculation. We use the angular frequency of bulk phonons in our model which has been found to be a good approximation for germanium nanowires with diameters above 10 nm [8].

Finally we come back to the *effective deformation potentials*, E_1 in Equation S10 and DK in Equation S12. E_1 describes the local energy shift of the valence band that is caused by the presence of a phonon distorting the crystal structure [13, 14]. The acoustic deformation potential is a second rank tensor quantity [13, 14], related to the strain tensor. For the valence band there are three deformation potentials [15] due to the fact that the valence band is derived from a *p*-orbital, that is, deformation potential related to (i) isotropic deformations, (ii) deformations along the [100] direction and (iii) deformations along the [111] direction. Despite this complexity it has been found that a single, scalar, effective deformation potential can be used which results in an error of less than 5% in the final calculation [16, 17]. Similarly the effective optical deformation potential DK should be a vector quantity that is related to the displacement of the atoms by the phonon [13, 14]. This too can be approximated by an effective scalar which has been shown to introduce a negligible error [16, 17].

Coulomb Scattering

The momentum relaxation time for a carrier in a subband i to scatter due to a Coulomb scattering centre located at r' is given by the following set of equations [18]:

$$\frac{1}{\tau_i^{(C)}} = \frac{\pi L}{2 g_v \hbar} \sum_{i'} (g_{i'}(E) \pm g_i(E)) I_{q;ii'}^{(C)}$$

$$I_{q;ii'}^{(C)} = 2\pi L \int_0^{\infty} dr' r' n_C(r') \left| V_{q,ii'}^{(C)}(r') \right|^2 \quad \text{(S13)}$$

$$V_{q;ii'}^{(C)}(r') = \frac{Ze^2}{2\pi L \epsilon} \int_0^{\infty} drr R_{i'}(r) R_i(r) G_{mq}(r, r')$$

where $q = k_i \pm k_{i'}$ is the change of the hole wavevector upon scattering (*i.e.* the momentum transfer), L is the channel length of the device, $n_C(r)$ is the density of Coulomb centres at the position r, Ze is the charge located at that position, $\epsilon = \epsilon_r \epsilon_0$ is the dielectric constant of the material and the $\pm$ sign refers to forward and backward scattering. $G_{mq}(r, r')$ is a Green's function. These equations can be used for scattering from Coulomb centres located anywhere in the channel or in the dielectric material around the channel. The form of the Green's function changes depending on the relative position of the scattering centre and the charge carrier. In our calculation we assume that all the traps are located at the core/shell interface, by setting $n_C(r) = n_{ss}\delta(r - R)$, where $\delta(r - R)$ is the delta distribution, and we assumed that all of the holes were located in the germanium core. In this case the Green's function is given by the following set of equations [4]:

$$G_{mq} = a_G I_m(qr)$$

$$a_G = b_G + \frac{c_G K_R}{I_0}$$

$$b_G = -\frac{f_G K_0}{I_0} - \frac{\epsilon_{Ge}}{\epsilon_{shell}} K_{r'} \quad \text{(S14)}$$

$$f_G = c_G - \frac{\epsilon_{Ge}}{\epsilon_{Shell}} I_{r'}$$

$$c_G = \frac{\epsilon_{Ge}(\epsilon_{Ge} - \epsilon_{Shell}) x_R I_R I_R' (I_{R'} K_0 - K_{r'} I_0)}{\epsilon_{Shell}(\epsilon_{Ge} - \epsilon_{Shell}) x_R I_R' (I_R K_0 - K_{R'} I_0) - \epsilon_{Shell}^2 I_0}$$

where $K_R = K_m(x_R)$, $K_{r'} = K_m(qr')$, $I_{r'} = I_m(qr')$, $I_R = I_m(x_R)$, $I_0 = I_m(q(R + t_{shell}))$, $K_0 = K_m(q(R + t_{shell}))$, $I_R' = dI_R/d(qr)$, $I_m(qr)$ and $K_m(qr)$ are modified Bessel functions of the first and second kind, respectively, $x_R = qR$, ϵ_{Ge} is the dielectric constant of germanium and t_{shell} is the thickness of the oxide. We note that if

$t_{shell} \rightarrow 0$ then the Green's function is zero at all points. We therefore must include the shell in the calculations. Equation S14 shows that the Green's function is modified by the thickness and dielectric constant of the shell.

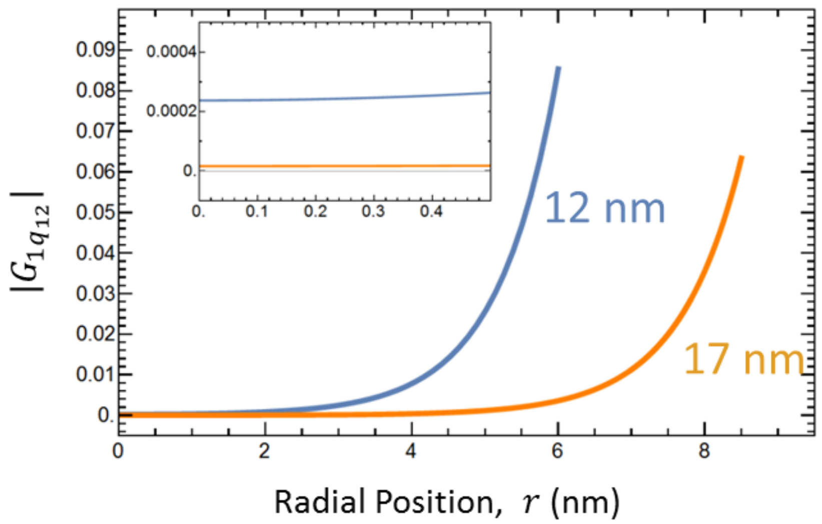

Figure S1: Green's function for Coulomb scattering as a function of radial position in the wire. The blue line corresponds to a wire with a diameter of 12 nm, while the orange line shows a 17 nm wire. The Greens function is larger for the smaller wire at all lengths. The insert shows a closer view of the Green's functions near to the centre of the wire. It is clear from these graphs that the scattering will depend very strongly on the distribution of the electrons within the wire. The value of the Green's function at the surface is 360 times larger than at the centre of the nanowire for the 12 wire and almost 4000 times larger for the 17 nm wire.

To demonstrate that the value of the Green's function is not strongly affected by the parameters thickness and dielectric constant of the shell, we examined the effect of changing them to a range of values. We assumed the value of the dielectric constant to be that of silicon, 11.9 [19], and that of germanium oxide, 7.4. The value of the Green's function was approximately 10 % lower of the case of the silicon compared to germanium oxide. A change of less than 1 % was seen when we changed the thickness of the shell from 20 nm to 1 nm. Therefore the choice of parameters here does not have a large bearing on the final result. For these reasons we took the dielectric constant to be the same as for germanium oxide and assumed a constant thickness of 1 nm.

In Figure S1 we show the Green's function for scattering from the first ($i = 1$) to the second ($i' = 2$) subband for a nanowire with a 12 nm diameter and a wire with a 17 nm diameter wire as a function of r, the radial position of the charge carrier in the

wire. The Green's function increases strongly approaching the surface of the wire, where the trapped charge is located, especially for the larger diameter wires. In this case the value of the Green's function at the surface ($r = R$) is 360 times larger than the value at the centre ($r = 0$) of the nanowire for the 12 nm wire, and 3958 times larger in the case of the 17 nm wire. For this reason the Coulomb scattering will be strongly affected by the distribution of the carriers within the wire, especially for larger diameter wires. We expect that at large diameters the charge carries will be located closer to the surface, and so scattering from electrons in the interface states will play a larger role. We do not take into account this carrier distribution and we are therefore likely to underestimate quantitatively the Coulomb scattering especially for large diameter wires.

In Table S1 the values of the parameters used in the calculation are shown. The calculation of the mobility involves solving the Kubo–Greenwood formula [6, 7], which contains an integral over the energy from zero to infinity. In principle we should therefore include infinitely many subbands. In practice however we solve this integral numerically and include only a limited number of subbands. Only holes near to the Fermi level contribute to the mobility as quantified (following Refs.[4] and [5]) by the term $1/(4k_BTcosh^2(\frac{E-E_f}{2k_BT}))$.

Table S1: Constants used in calculating the mobility

Symbol	Description	Value	Unit
α	Non-parabolicity parameter	0.7 for heavy holes; 0.2 for light holes [10]	eV^{-1}
U_i	Total potential energy of the subband i	0	eV
m_{HH}^*	Effective mass of the heavy holes at $k = 0$	$0.28m_0$ [20]	kg
m_{LH}^*	Effective mass of the light holes at $k = 0$	$0.044m_0$ [20]	kg
E_1	Average acoustic deformation potential	6.49 [17]	eV
DK	Average optical deformation potential	12.17×10^8 [17]	eV/cm
$\hbar\omega_{op}$	Energy of optical phonon	37.04 [17]	eV
u	Speed of sound in germanium	5400 [17]	m/s
ϱ_{Ge}	Density of germanium	5.32 [17]	g/cm^3
ϵ_{shell}	Dielectric constant of the shell	7.4	
n_{ss}	Density of surface states	10^{13}	cm^{-2}

Using the Fermi level determined from Equation S8 we find the first subband for which this term is below 10^{-3} for all energies and for every wire radius, which we refer to as the top subband. All subbands up to and including the top subband are included in the calculation. This is done for both light- and heavy-hole subbands. The number of subbands used in the calculation is diameter dependent both due to the

diameter dependence of the Fermi level and the diameter dependence of the intersubband energy spacing.

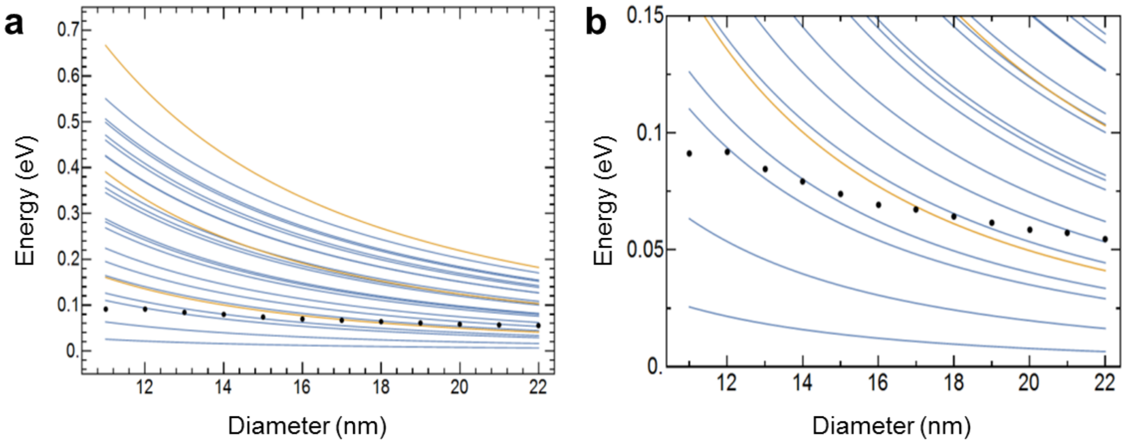

Figure S2: Position of subband minima and Fermi level as a function of nanowire diameter. The blue lines correspond to heavy-hole bands, and the orange lines represent light-hole bands. The black dots are the Fermi level positions for the diameters at which the calculation was carried out. (a) and (b) show the results of the same calculation, but with different energy scales. (a) shows a broader energy scale to emphasise the spreading of the subband bottoms as the nanowire diameter is decreased, and the disparity in the energy separation of the heavy-hole and light-hole subbands. (b) shows the position of the Fermi level relative to the subband minima.

In Figure S2a we plot the position of the bottom of the first 20 heavy-hole bands, in blue, and the first 3 light hole bands, in orange. It becomes apparent that the energy separation between the bottoms of the bands is increased as the diameter is decreased. Although we have plotted the bottom of the subband ($k = 0$) here, the same behaviour is seen for all values of k. Figure S2b shows the same evolution of the band bottoms as Figure S2a, but with a smaller energy scale to better show the change of the Fermi level at different diameters. It should be noted that in Figure S2b the scale on the *y*-axis is in steps of 25 meV, which is the thermal energy at room temperature, and therefore provides insight into the diameter at which single subband effects may be occurring.

The mobility of light and heavy holes, μ_{LH} and μ_{HH} respectively, will be different due to their dissimilar effective masses. We therefore calculate the mobility of light and heavy holes separately and then find the total mobility μ_{tot} :

$$\frac{1}{\mu_{tot}} = \frac{1}{\mu_{HH}} + \frac{1}{\mu_{LH}}. \tag{S15}$$

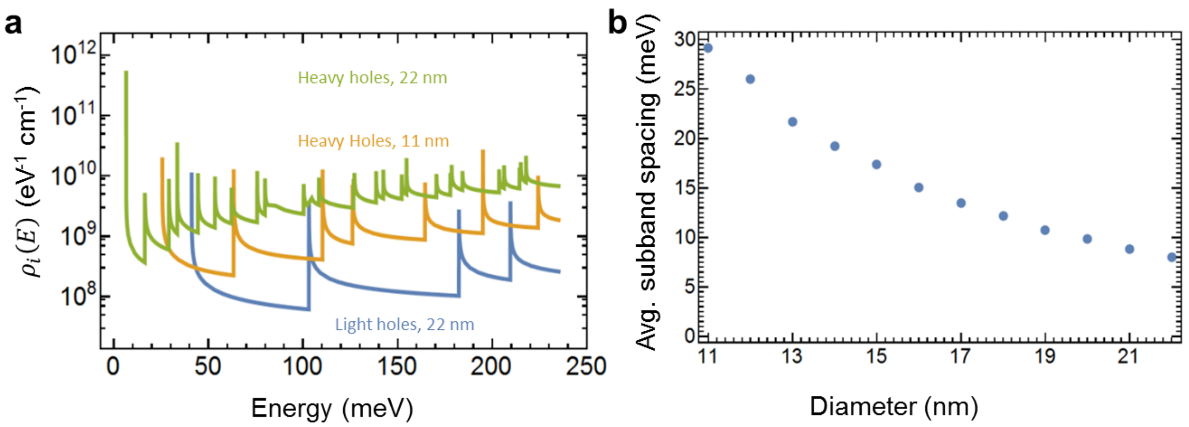

Figure S3: (a) Density of states and (b) the average subband bottom's spacing as a function of wire diameter. (a) shows the density of states for heavy holes and light holes in a 22 nm diameter wire, and the density of states for heavy holes in an 11 nm wire. The density of states for the heavy holes in the thinner wire is about an order of magnitude less than in the thicker wire. This reduction in the density of states is one of the factors that leads to a diameter dependent resistivity. Similarly the density of states for light holes in a wire of the same diameter is 1.5 to 2 orders of magnitude lower than that of the heavy holes. It is for this reason that heavy holes dominate charge transport properties. Each of the spikes is caused by the singularity at the bottom of each one-dimensional subband. (b) shows the average subband bottom spacing with all the subbands used in the calculation taken into account with equal weight. It can be seen that the average spacing is comparable to the thermal energy at room temperature, suggesting that the use of the one dimensional Kubo–Greenwood formula is justified.

Scattering into light-hole and heavy-hole bands is taken into account in both cases. In general the light holes will have a higher mobility due to their lower effective mass, however the mobility of the material will be mainly determined by the heavy holes due to their greater density of states [10]. This is shown in Figure S3a where we plot the density of states for light and heavy holes in a nanowire with a 22 nm diameter. It can be seen that over the entire energy range the density of states of the heavy holes is 1 to 2 orders of magnitude larger than the light holes. This is due to both the fact that the density of states in a single band is higher, as well as from the fact that at any given energy there will be more heavy-hole bands contributing to the density of states due to the smaller energy separation between heavy-hole bands. The position of the Fermi level at each diameter is indicated in Figure S3a by the position of the black dots, and it is clear that there are a greater number of heavy hole bands beneath the Fermi level at every diameter. It has been found that in germanium at

220 K 96 % of the holes are heavy, with 4 % light [10] [2], and remains unaffected down to 40 K. We therefore expect that there should not be a substantial change at room temperature.

The average subband spacing for different wire diameters was found using the expression:

$$\frac{1}{\eta_{top\ subband}(R)} \sum_{i=1}^{top\ subband} E_{i+1}(R,0) - E_i(R,0) \qquad \text{(S16)}$$

where $E_i(R,0)$ is the energy of the bottom of the subband i for a wire of radius R and $\eta_{top\ subband}(R)$ is the number subbands used at that radius. It is plotted in Figure S3b. It is evident that the average spacing is comparable to the thermal energy at room temperature, 25 meV. The subband spacing close to the Fermi level exceeds 25 meV for the smallest diameter wires that we consider, and there are only a few subbands within a k_BT of the Fermi level at all diameters. We note that the one dimensional Kubo–Greenwood formula has previously been used when the average subband spacing was found to be 10 meV [5]. Therefore the use of the one dimensional formula is reasonable.

Form factor

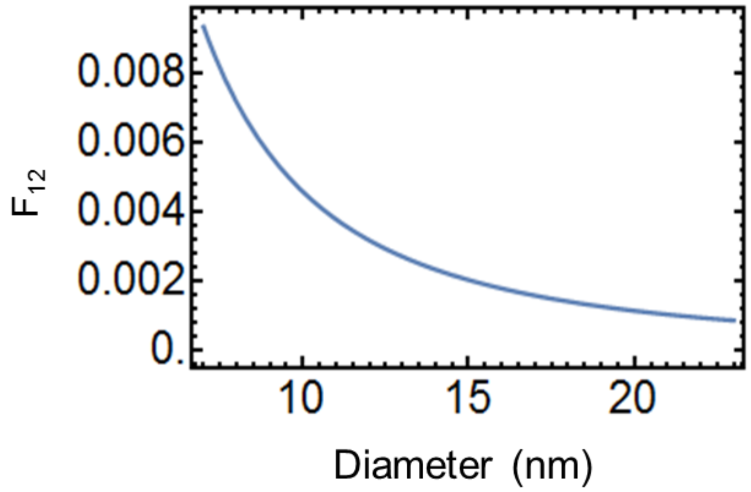

Figure S4: Form factor (Equation S11) for scattering from the first to the second subband.The fact that the form factor increases as the radius of the nanowire is decreased is clearly seen. This stems from the normalisation prefactor, $\sqrt{2}/RJ_{m+1}(j_{mn})$ in Equation S7. This impacts on the diameter dependence of the resistivity, as it results in the decrease in the phonon-limited mobility as the wire radius is decreased.

[2] The population of the split-off band was found to be negligible in the same study. In addition our Fermi level was found to be at most 92 meV, far below the split off distance of 296 meV [17] . For both of these reasons we do not include the split-off band in our calculations.

Width of the space charge region $\boldsymbol{d}$

Inserting Equation S4 into Equation S2 we can find the width of the space charge region d for a given nanowire radius R, electrostatic surface-potential φ_0 and ϵ_r. We assume that the relative permittivity is the same as in bulk, 16 for the case of germanium [19]. ϕ_0 has been found to be 0.3 eV for *p*-type germanium nanowires with a native oxide [3]. In Figure S5 we plot the ratio of d to R as a function of nanowire diameter. We have assumed that $n_{ss} = 10^{13}$ cm^{-2}. We note that $R \approx d$ for a wire with a diameter of about 18 nm.

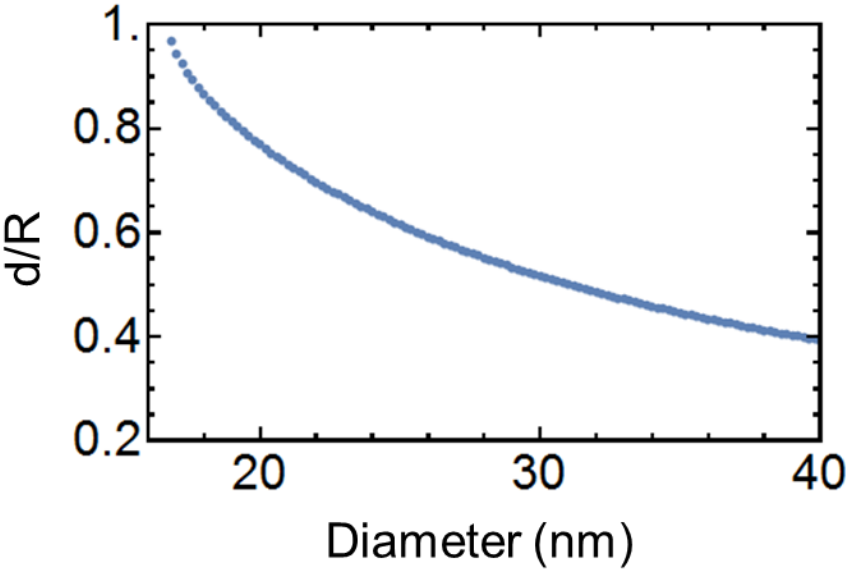

Figure S5: Ratio of space charge region width d to nanowire radius R as a function of diameter by solving the Poisson equation. The density of surface states is assumed to be 10^{13} cm^{-2}. The mobile holes are distributed in an annular region near to the edge of the nanowire for nanowires with diameters above ≈ 18 nm.

References:

1. Bardeen, J. *Phys. Rev.,* **1947**, *71*, 717-727.
2. Cardona, M.; Peter, Y. Y. *Fundamentals of semiconductors*, Springer, 2005.
3. Wang, D.; Chang, Y.-L.; Wang, Q.; Cao, J.; Farmer, D. B.; Gordon, R. G.; Dai, H. *J. Am. Chem. Soc.,* **2004**, *126*, 11602-11611.
4. Jin, S.; Fischetti, M. V.; Tang, T.-W. *Appl. Phys. Lett.,* **2007**, *102*, 083715.
5. Dura, J.; Triozon, F.; Barraud, S.; Munteanu, D.; Martinie, S.; Autran, J. *J. Appl. Phys.,* **2012**, *111*, 103710-103719.
6. Kotlyar, R.; Obradovic, B.; Matagne, P.; Stettler, M.; Giles, M. *Appl. Phys. Lett.* **2004**, *84*, 5270-5272.
7. Kubo, R. *J. Phys. Soc. Jap.,* **1957**, *12*, 570-586.

8. Greenwood, D. *Proc. Phys. Soc.,* **1958**, *71*, 585.
9. Hattori, J.; Uno, S.; Nakazato, K.; Mori, N. *J. Appl. Phys*., **2010**, *107*, 033712-033717.
10. Godoy, A.; Yang, Z.; Ravaioli, U.; Gámiz, F. *J. Appl. Phys*., **2005**, *98*, 013702-013705.
11. Rodríguez-Bolívar, S.; Gómez-Campos, F.; Gámiz, F.; Carceller, J. *J. Appl. Phys*., *2005*, *97*, 013702-013710.
12. Ashcroft, N. W.; Mermin, N. D. *Solid State Physics*, Holt, Rinehart and Winston, New York, 1976.
13. Ferry, D. K.; Goodnick, S. M. *Transport in nanostructures*, Cambridge University Press, 1997.
14. Ferry, D. K. *Semiconductor transport*, CRC Press, 2000.
15. Ridley, B. K. *Electrons and phonons in semiconductor multilayers*, Cambridge University Press, 2009.
16. Bir, G.; Pikus, G. *Sov. Phys. Sol. State* **1961**, *2*, 2039-2051.
17. Lawætz, P. *Phys. Rev*., **1968**, *174*, 867.
18. Fischetti, M. V.; Laux, S. E. *J. Appl. Phys*., **1996**, *80*, 2234-2252.
19. Lee, J.; Spector, H. N. *J. Appl. Phys*., **1983**, *54*, 3921-3925.
20. Sze, S. M. *Physics of Semiconductor Devices*, Wiley, New York, 1981.
21. Jacoboni, C.; Reggiani, L. *Rev. Mod. Phys*., **1983**, *55*, 645.
